# Evaluation of Cytocompatibility of PEEK-Based Composites as a Function of Manufacturing Processes

**DOI:** 10.3390/bioengineering10111327

**Published:** 2023-11-17

**Authors:** Jorge Gil-Albarova, María José Martínez-Morlanes, José Miguel Fernández, Pere Castell, Luis Gracia, José Antonio Puértolas

**Affiliations:** 1 Department of Surgery, Universidad de Zaragoza, 50018 Zaragoza, Spain; 2 Instituto de Investigación en Ingeniería de Aragón, I3A, Universidad de Zaragoza, 50018 Zaragoza, Spain; mjm230181@gmail.com (M.J.M.-M.); lugravi@unizar.es (L.G.); japr@unizar.es (J.A.P.); 3 Department of Materials Science and Technology-EINA, Universidad de Zaragoza, 50018 Zaragoza, Spain; 4 AITIIP Technological Center, 50720 Zaragoza, Spain; josemiguel.fernandez@aitiip.com (J.M.F.); perecastell@gmail.com (P.C.); 5 Department of Mechanical Engineering-EINA, Universidad de Zaragoza, 50018 Zaragoza, Spain

**Keywords:** PEEK, polyetheretherketone, composite, graphene, cytocompatibility, bioactivity, fused filament fabrication, FFF, 3D printing

## Abstract

The biocompatible polymer polyetheretherketone (PEEK) is a suitable candidate to be part of potential all-polymer total joint replacements, provided its use is associated with better osseointegration, mechanical performance, and wear resistance. Seeking to meet the aforementioned requirements, respectively, we have manufactured a PEEK composite with different fillers: carbon fibers (CF), hydroxyapatite particles (HA) and graphene platelets (GNP). The mechanical outcomes of the composites with combinations of 0, 1.5, 3.0 wt% GNP, 5 and 15 wt% HA and 30% of wt% CF concentrations pointed out that one of the best filler combinations to achieve the previous objectives was 30 wt% CF, 8 wt% HA and 2 wt% of GNP. The study compares the bioactivity of human osteoblasts on this composite prepared by injection molding with that on the material manufactured by the Fused Filament Fabrication 3D additive technique. The results indicate that the surface adhesion and proliferation of human osteoblasts over time are better with the composite obtained by injection molding than that obtained by 3D printing. This result is more closely correlated with morphological parameters of the composite surface than its wettability behavior.

## 1. Introduction

Intrinsic characteristics of poly (ether-ether-ketone) (PEEK), such as high mechanical and tribological performance, biocompatibility and thermal and chemical stability, which withstand sterilization by steam or gamma irradiation [1], have led to its implementation as a biomaterial in several clinical applications related to orthopedic spine and trauma implants [2,3]. A major drawback of metallic components in total joint replacements is the release of harmful metal ions which increase the presence of metallic ions in human blood. In line with this, some current research in the former fields is focused on the development of an all-polymer implant. PEEK is the main candidate to replace metallic components in total joint prostheses due to its aforementioned good properties. Nonetheless, PEEK needs to meet three conditions for achieving this goal: it should promote good osseointegration, improve the mechanical strength for load bearing and decrease wear in comparison to the counterpart in bearing pairs. In order to achieve these objectives, neat PEEK must be reinforced with different fillers with specific functions, such as we will see below.

Concerning the weak interaction of PEEK with bone, several strategies have been developed to enhance the fixation of PEEK implants to bone. The creation of porous networks, which allow bone ingrowth, is one of the main mechanisms to be tested. Several porous structures with different geometries and pore sizes have been manufactured on the surface of metallic orthopedic implants by thermal spray coating or sinterization, among other methods, to mimic the morphology of trabecular bone. However, other methods must be used for creating scaffolds for cell attachment with a suitable vascularization when the substrate is a polymer like PEEK. Additive manufacturing is an alternative and more sustainable method to fabricate scaffold than traditional manufacturing processes, since it allows complex and controlled geometries to be obtained with a high degree of design freedom, by the creation of an implant from deposited or fused material layer by layer, which could facilitate the fabrication of customized prostheses. Various 3D-printing techniques have been used to obtain PEEK and reinforced PEEK, Fused Filament Fabrication (FFF) being the most common method for obtaining the layers. The lack of homogeneity in the specimen due to intra- and inter-layer defects generates hierarchical [4] porosity, which is partially eliminated with surface or thermal treatments [5,6]. On the other hand, this technique also makes it possible to obtain controlled porosity with total connectivity [7,8]. Scaffolds have also been obtained by other additive techniques, such as Selective Laser Sintering (SLS) [9] and Selective Laser Melting (SLM) [10].

Besides the osteoconduction that porosity provides, to achieve good osseointegration, it is necessary to reduce the bioinert character of PEEK. The addition of hydroxyapatite (HA) micro or nanoparticles particles helps to achieve the aforementioned goal, as HA confers a remarkable ability to bind to bone [11]. The bioactivity of this HA/composite has been assessed in some studies with positive results, HA being found to provide a more favorable environment for bone ingrowth than unfilled PEEK [12], and positive cell attachment, proliferation, spreading and alkaline phosphatase (APL) activity were observed to be higher than in pristine PEEK [13,14]. In this context, new PEEK materials have appeared on the market, such as VESTAKEEP PEEK and PEEK^MBT^, which enhance the osteoconductivity of PEEK [15].

To fulfil the second condition, i.e., increase the mechanical performance of PEEK, the strategy has consisted of reinforcing PEEK with a range of fillers. This approach enhances the elastic modulus of unmodified PEEK; its value is still similar to that of bone and the stress-shielding effect remains. The main fillers used have been glass fibers [16], carbon fibers (CFs) [17] and, more recently HA microparticles up to 30 vol% [18] or smaller amounts of nanohydroxyapatite (nHA) [19], this material having the additional function of enhancing the bioactivity of PEEK, as described in the previous paragraph. Additionally, some authors have tested new fillers such as carbon nanotubes (CNTs) or graphene due to their extremely high elastic modulus, close to 1 TPa, and tensile strength, 130 GPa [20]. In general, the outcomes show significant increases in the elastic modulus, which can reach twice that of the trabecular bone, but with the drawback of a loss of toughness. Various new attempts to address the composite’s weakness include the preparation of hybrid composites with HA and CF prepared by a mechano-fusion process [21], with nHA and carbon nanofibers, [19] or graphene nanosheets and CNTs [9].

The two-dimensional structure of graphene provides not only large surface areas in contact with the matrix, which improves the strength of the composites, but may also act as a solid lubricant, potentially enhancing the tribological response in bearing pairs [22]. In this research article, Puértolas et al. demonstrate that graphene nanoplatelets have lubricant properties (GNP) in PEEK with a composition between 1 and 10 wt%. The nanocomposites were obtained by solvent-free melt blending and injection molding. Strong reductions in wear factor (−83%) and coefficient of friction (−38%) were observed at the maximum GNP reinforcements.

Several methods [23,24,25] have been used to improve both the bioactivity and the strength of PEEK-based materials, such as the combination of PEEK and cellular calcium hydroxyapatite (CHAp), constructions using extrusion methods, composite surface coatings, surface modifications, or multilayer structures. In relation to this, the use of MC3T3-E1 cells cultured in DMEM culture medium supplemented with 10% fetal bovine serum, 1% penicillin and streptomycin sulfate demonstrated in vitro that cell attachment, proliferation and spreading, as well as APL activity, were higher in the HA/PEEK composite than pure PEEK or ultra-high molecular weight polyethylene [14]. Cultured human osteoblast-like MG63 cells and murine fibroblast L929 cells obtained from ATCC were used for cytocompatibility testing, demonstrating that the PEEK composite promoted cell attachment and proliferation, and osteogenic differentiation, as well as being associated with higher ALP activity and increased calcium nodule-formation, compared with pure PEEK [26]. Wang et al. [27] reported that Sr/Si ion surface modification has a stronger osteo-inductive effect on the bone marrow-derived stem cells (BMSCs) derived from ovariectomized rats, improving PEEK osteointegration. Moreover, human umbilical vein endothelial cell-derived decellularized extracellular matrix could be also an option to mediate endothelial cell-osteoprogenitor crosstalk to promote a favorable microenvironment for scaffold osteointegration, as in PCL [28].

The aim of this study is to assess the influence of the manufacturing process on the cell viability of human osteoblasts on two PEEK hybrid composites reinforced with CFs, HA, and graphene nanoplatelets (GNPs) in percentages which have optimized the mechanical properties and wear resistance and contribute to obtaining a good interaction with bone. One of the composites was fabricated by injection molding (IM) and the other by 3D printing, using a new additive manufacturing method based on FFF 3D printing which is considered a useful tool for obtaining customized implants and also is a method that introduces porosity into a specimen.

## 2. Materials and Methods

### 2.1. Materials and Fillers

PEEK grade 90P was provided in pellets by Victrex plc, Thornton-Cleveleys, UK, with a glass transition temperature of 143 °C, melting temperature of 345 °C, and density at 25 °C of 1.30 g/cm^3^. PEEK grade 150CA30 was also provided in pellets by Victrex plc, UK, with a glass transition temperature of 143 °C, melting temperature of 343 °C, and density at 25 °C of 1.40 g/cm^3^, which were dried at 150 °C before use for the production of the nanocomposites. Both grades were dried at 150 °C for 4 h before use. Pristine GNPs (avan-GRAPHENE) were provided in powder form by Avanzare Innovación Tecnológica, SL (Navarrete, Spain), and HA was purchased from Sigma Aldrich (Burlington, MA, USA) in powder form several microns in size.

### 2.2. Composite Materials

Composites were denoted in this study with three digits corresponding to the weight percentages of the three potential reinforcement materials, CFs, HA and GNPs, followed by the term -IM for the samples manufactured by injection molding and -3D for the printed samples: xxCF-xxHA-xxGNP/PEEK-IM or xxCF-xxHA-xxGNP/PEEK-3D (Table 1). Composites were prepared by GNP concentrations 0-0-xx/PEEK with xx = 0, 1.5, 3.0, and 4.5 wt%, hydroxyapatite particles 0-xx-0/PEEK with xx = 0, 5, 15 wt% and 30 wt% of carbon fibers, 30-0-0/PEEK. Some combinations were also manufactured, like 0-5-3/PEEK and mainly the 30-8-2/PEEK, which is the object of the cytocompatibility study. The reasons for choosing this last formulation, 30-8-2/PEEK, will be discussed in the further paragraphs. For simplicity, the composites with the last composition will be referred to as IM and 3D as a function of the used processing technique.

### 2.3. Processing Technologies

#### 2.3.1. Extrusion Compounding

PEEK nanocomposites were prepared with a 26-mm diameter co-rotating twin-screw extruder, L/D = 40, (Coperion ZSK 26 Mc18) by melting the polymer and mixing the corresponding additives dosed with a Coperion K-TRON gravimetric feeder specifically designed to work with powder and fibers. The temperature profile set in the extruder was optimized in previous studies and was set from 330 °C in the feeder, increasing by 5 °C in each screw element (with a total of 11 in the screw) up to 390 °C in the die. The temperature of the polymer was higher than its melting point due to the shear forces applied during processing. A high shear rate screw profile, which was specifically designed for this process, was used to ensure proper dispersion of the selected additives. The rotor speed was set to the same value (250 rpm) for processing all the nanocomposites. Three feeders were required for this process: one main feeder for feeding PEEK at a speed of 10 kg/h and two lateral ones for feeding graphene and HA at feeding positions 4 and 10 of the barrel. The nanocomposites were dried at 150 °C directly after pelletization and stored in sealed bags.

#### 2.3.2. Injection of Specimens for Mechanical Characterization

Dog-bone shaped specimens (type 1BA) for tensile tests, under ISO 527 [29], and specimens for flexural tests (80 mm × 10 mm × 4 mm), under ISO 178 [30], were injected into a mold made of tool steel (1.2790) at a constant temperature of 180 °C. The pellets were dried at 150 °C for 3 h before processing. The specimens were produced by IM through a JSW 85 EL II injection machine with a 35-mm diameter reciprocating screw at a screw speed of 120 rpm with a temperature profile from 340 °C in the hopper up to 375 °C in the dye. The dosage used in each specimen was 20 mm with an injection time of 0.36 s, the compaction pressure was 1000 bar, and the cooling time was 3 s. Unfilled PEEK and the specimens that contain different loadings of GNPs and HA were prepared following this protocol. At least 15 samples were produced for each composition.

#### 2.3.3. Filament Preparation

Filaments for FFF processing of 1.75 mm diameter were prepared in a COMPOSER 450 filament maker purchased and upgraded by 3DEVO (Utrecht, The Netherlands). The temperature profile was set from 350 to 390 °C, the screw speed at 5 rpm and the nozzle extruder at 1.5 mm. Spools of the filaments were obtained with the required compositions and stored before use. 

#### 2.3.4. Filament Fused Fabrication 

Specimens for mechanical characterization following ISO standards were produced by FFF using an INTAMSYS Funmat HT printer. Specifically, 3D 5 × 5 × 2 mm samples were fabricated with the same printing technique, while samples with the same dimension were cut from the injection specimens. The conditions used for printing the samples were a nozzle temperature of 400 °C and a chamber temperature of 156 °C. 

### 2.4. Surface Morphology 

The morphological surface characterization of both types of manufactured composites was performed using a confocal microscope Sensofar PLm 2300 optical imaging profiler (Sensofar, Barcelona, Spain).

Scanning electron microscopy (SEM) images of the surfaces were taken with a Gemini Field Effect Scanning Electron Microscope (Carl Zeiss, Hombrechtikon, Switzerland) in the secondary electron mode at 5 keV and with a probe current of 100 pA. All samples were examined after Au coating using a sputtering device Balzers-SCD-4 (BAL-TEC, Balzers, Liechtenstein).

Surface wettability was measured by an Attension^®^ Theta tensiometer (Biolin Scientific, Gothenberg, Sweden). The contact angle was determined by measuring the static water contact angle using the sessile drop method. The volume of the deionized water drop was 0.1 μL. Three specimens were used for each composite.

### 2.5. Mechanical Properties

Mechanical properties were analyzed using tensile and bending tests. The mean and standard deviation values for the mechanical parameters were acquired with a sample size of n = 3. Tensile tests were performed according to [29] using a universal testing machine Instron 5565, at a speed of 10 mm/min. Three-point bending tests were performed according to [30], with a speed of 2 mm/min. For this testing, IM was used to prepare samples of PEEK, extruded PEEK and PEEK reinforced with GNPs at different concentrations of 1.5, 3.0 and 4.5 wt%.

Hereafter, to analyze the effect of HA on PEEK, two different formulations with HA concentrations of 5 and 15 wt% also were prepared by IM and tested. Finally, as a decrease in mechanical properties is foreseen with HA addition to virgin PEEK, a concentration of 5.0% HA was chosen as appropriate to obtain the composite material GNP-HA/PEEK. Hence, samples corresponding to 5.0 wt% of HA and 3.0 wt% of GNPs were prepared and tested.

After observing the influence of GNP and HA on the mechanical properties of the PEEK matrix separately, a combination of both fillers was chosen to produce 0-5-3/PEEK. This choice of 3 wt% GNP was based on the mechanical properties and also on the influence of the GNP content on the wear rate and the coefficient of friction with a reduction of 44% and 23%, respectively, according to [22]. Since the mechanical strength of the former composite was not satisfactory to meet the loading requirements of potential implants, we decided to incorporate 30 wt% of carbon filler, which is a common percentage in industrial reinforced PEEKs. However, to reduce economic cost and time, we decided to incorporate this fiber content with 8 wt% HA and 2 wt% GNP. The increase in HA from 5 to 8 wt% was in line to increase the osseointegration of the composite, while the reduction in GNP from 3 to 2 wt% was to reduce the loss in toughness while maintaining the lubricating ability of GNP in the matrix according to [22]. This 30-8-2/PEEK composite was used in the bioactivity study. Mechanical properties were measured prior to sterilization.

### 2.6. Cytocompatibility

The cell viability of human osteoblasts cultured for 1, 4 and 7 days on IM and 3D samples of polyetheretherketone composites were studied. Likewise, the morphology and organization of the actin cytoskeleton in cells cultured for 7 days on said samples were also examined.

### 2.7. Composite Materials for Bioactivity Studies

The samples used were the aforementioned 2 mm thick squares (5 × 5 mm) of 30-8-2/PEEK obtained by 3D printing or IM. The samples were sterilized using a Steris pressure cooker autoclave, specifically the Amsco Century V-1262 model^®^. The process involved heating to 134 °C for 6 min under a pressure of 2 atmospheres and a humidity level of 3%.

### 2.8. Cell Type

Primary cultures of human osteoblasts were established from trabecular bone from bone explants from three patients with primary osteoarthritis, ranging between 60 and 75 years of age, who underwent hip arthroplasties. To carry out the experiments, phase 1 cultures were used. The phenotypic characterization of osteoblast cultures has been demonstrated in previous publications [31].

### 2.9. Assays for Changes in Osteoblast Viability

Three experiments were carried out, each with cells from different patients and using two samples corresponding to each material (Table 2).

Approximately 5 × 10^3^ cells were seeded on the samples deposited in 48-well plates in a final volume of 0.5 mL of DMEM medium supplemented with 15% fetal bovine serum and antibiotics and incubated for 1, 4 or 7 days. As a control, a sample of each type of processing was incubated only with culture medium and subjected to the same experimental treatment. Specific devices were used to keep the materials at the bottom of the well and prevent them from floating in the culture medium. In all cases, the disks were transferred to sterile wells after 1 day of culture to exclude possible interference from cells adhering to the culture plastic during seeding. 

After the incubation periods, the samples were washed with phosphate-buffered saline (PBS) and incubated for 4 h in culture medium containing 10% Alamar Blue, a commercial reagent that detects mitochondrial activity. The fluorescence emitted was quantified using a spectrofluorometer. The fluorescence signal of samples incubated with cells was subtracted from that of samples incubated without cells. At the end of the incubation period, cells were gently rinsed away with PBS to eliminate any unattached cells, and the viability of the attached cells was quantified. For technical simplicity, the alamarBlue assay (Biosource, Nivelles, Belgium), a redox indicator which changes color in response to metabolic activity, was used. This dye is generally used to measure the viability of a given cell population, and the assay can be used to measure quantitatively an increase in cell number as a function of cell viability. Preliminary experiments indicated that the alamarBlue assay and microscopic counting of viable cells using a trypan blue assay yielded almost identical results. Briefly, cells were incubated in DMEM containing 10% alamarBlue dye and, after excitation at 530 nm, the fluorescence emitted at 590 nm was quantified using a spectrofluorimeter (Synergie4, Evry, France) [31].

### 2.10. Assays of Cell Morphology and Organization of the Actin Cytoskeleton

Three experiments were carried out using the same cells as in the cell viability assays. One sample was used for each condition (Table 3).

The cells were seeded on the samples as described previously and incubated for 7 days. For visualization of the actin cytoskeleton, cells adhered to the materials were fixed with 4% paraformaldehyde in phosphate-buffered saline (PBS) and permeabilized with 0.1% Triton X-100 in PBS. Subsequently, the cells were incubated with phalloidin conjugated with tetramethyl-rhodamine isothiocyanate dissolved in PBS at 4 × 10^−7^ M. After two washes with PBS, to observe the cell nucleus, the samples were incubated with 4, 6 diamino-2-phenylindole dissolved in PBS at 3 × 10^−6^ M and examined using a confocal microscope.

### 2.11. Statistical Analysis

The graphs show the mean ± standard deviation (SD). Statistical analysis was carried out using SPSS, Version 25.0. Quantitative data were analyzed using Student’s t test after confirming that they followed a normal distribution using the Shapiro–Wilk and Kolmogorov–Smirnov tests. *p* values less than 0.05 were considered statistically significant.

## 3. Results

### 3.1. Surface Characterization 

**Figure 1:**
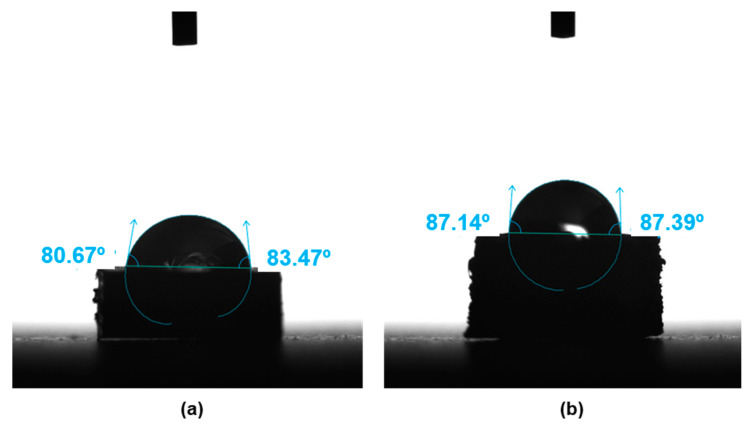
Contact angle for the 30CF-8HA-2GNP/PEEK composite obtained by: (**a**) injection molding; (**b**) 3D printing.

Figure 1 shows one of the three water drops and the respective contact angle in one specimen of each different manufactured composite: 30-8-2/PEEK-IM and 30-8-2/PEEK-3D, since we want to assess the cell viability only in these composites. The surface wettability analysis found contact angles of θ = 79.7 ± 5.3° for the composite manufactured by IM and θ = 85.7 ± 6.9° for the 30-8-2/PEEK composite produced by the FFF 3D printing technique. The result points out that this difference is not statistical relevant.

**Figure 2:**
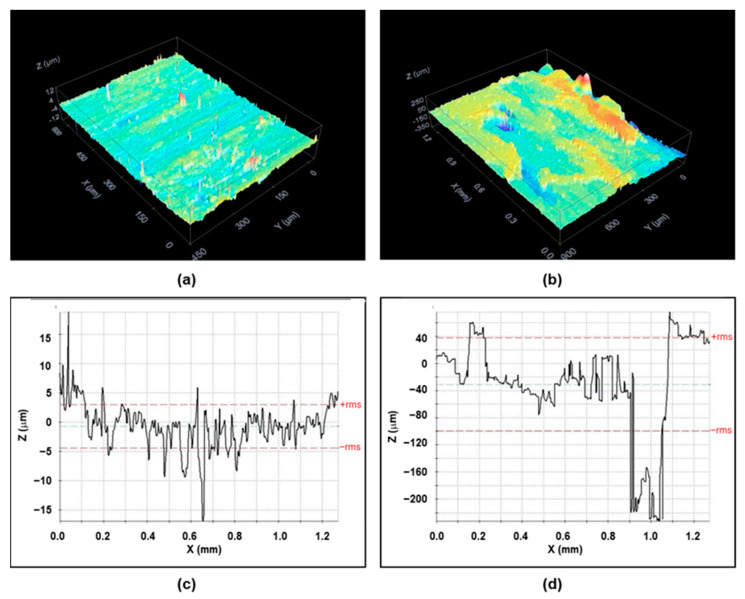
Confocal images and surface profiles of the 30-8-2/PEEK composites: (**a**) confocal image for injection molding; (**b**) confocal image for 3D printing; (**c**) surface profile for injection molding; (**d**) surface profile for 3D printing. Red dashed lines correspond to root mean square.

Concerning roughness behavior, the surface topography showed strong differences between the plaques obtained by IM and the FFF process, as we can observe in Figure 2. The confocal microscopy images gave an average roughness of R_a_ = 2.3 ± 3.2 μm in the 30-8-2/PEEK-IM composite, and one order of magnitude higher, R_a_ = 53 ± 72 μm, in the 30-8-2/PEEK-3D composite. Figure 2 also shows cross-section profiles illustrating some differences between the two composites. In the IM samples, the profiles display a homogeneous structure of peaks and valleys with a period of around 20–30 nm. In contrast, in the 3D samples, two clearly different zones are observed: one with a similar periodic structure to that in the IM composite, though with higher peaks and valleys, this corresponding to greater roughness, but also some rectangular valleys from 100–150 nm wide and 100–150 μm deep.

**Figure 3:**
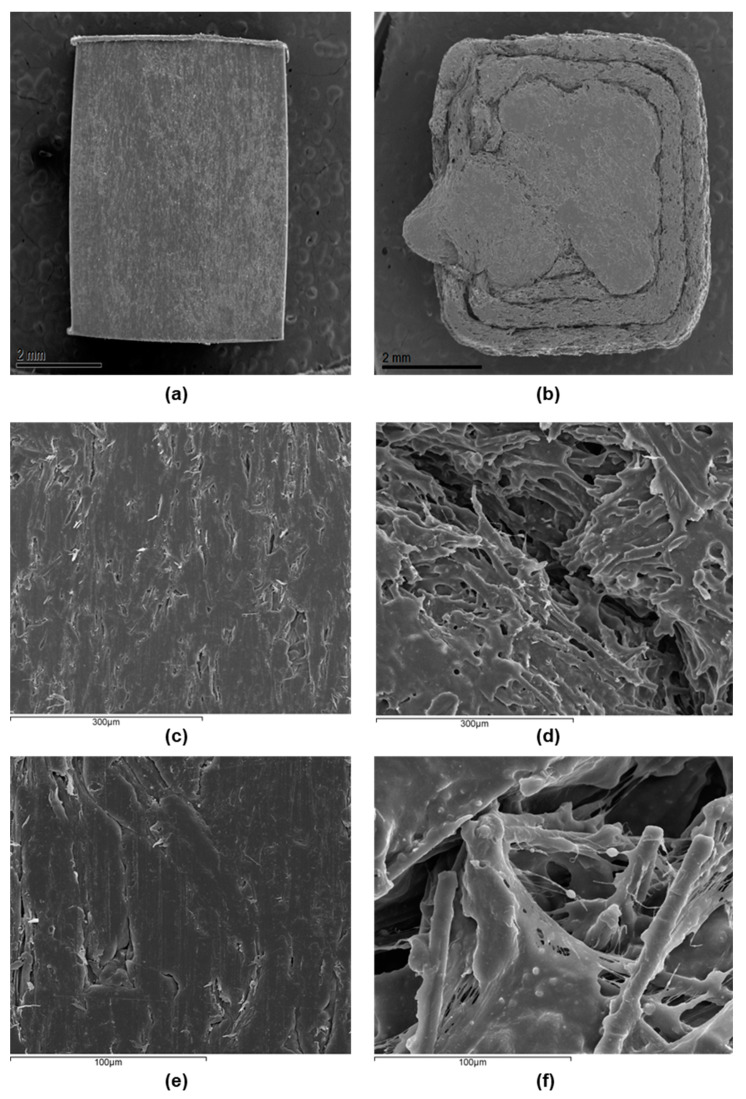
Samples and scanning electron microscopy images at different magnifications of the surface: (**a**) sample of injection molding composite; (**b**) sample of fused filament 3D-printing composite; (**c**) electron microscopy image for injection molding composite at 300 μm scale; (**d**) electron microscopy image for fused filament 3D-printing composite at 300 μm scale; (**e**) electron microscopy image for injection molding composite at 100 μm scale; (**f**) electron microscopy image for fused filament 3D-printing composite at 100 μm scale.

SEM images corroborate the former confocal information about the surface topography. Figure 3 illustrates the appearance of the 30-8-2/PEEK-IM composite surface, which is smooth at first sight but reveals small holes at high magnifications. The images of the 3D-printed composite surface show homogeneous regions with similar characteristics to those of IM samples, but these are separated by major cracks, consistent with the wide and deep valleys observed by confocal microscopy. At high magnifications, inside the cracks we can observe the presence of CFs embedded into PEEK and small particles several microns in size, which could correspond to HA particles. On the other hand, the SEM images did not detect any presence of GNPs because the magnifications used were very low for their observation, such as was detected in [22].

### 3.2. Mechanical Properties

**Figure 4:**
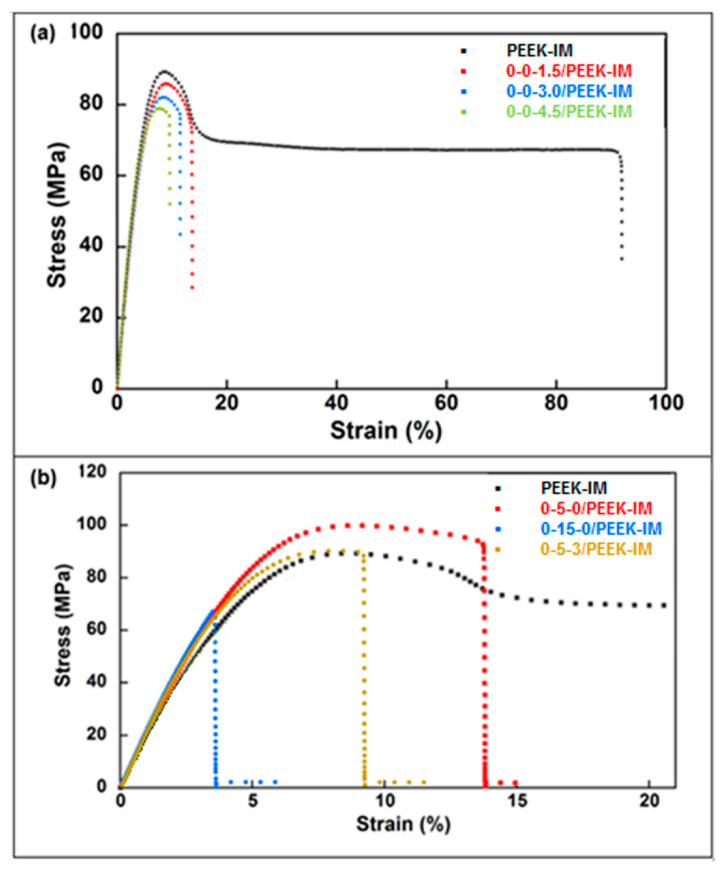
Tensile curves for samples prepared by injection molding compared with virgin and extruded PEEK. (**a**) Influence of graphene nanoplatelet (GNP) wt% concentrations: 0-0-xx/PEEK-IM. (**b**) Influence of hydroxyapatite (HA) wt% concentrations: 0-xx-0/PEEK and a composite manufactured with both kinds of filler (HA and GNP) at fixed concentrations: 0-5-3/PEEK. In all cases, the composites do not contain carbon fiber reinforcement.

Figure 4 and Table 4 show the results obtained in tensile tests. Results indicate an increase of 8.3% in Young’s modulus for higher GNP concentrations with respect to extruded PEEK, while it remains practically constant for intermediate GNP content. Tensile strength drops with the presence of GNPs, reaching a decrease of 11.1% at 4.5 wt% of GNP. The addition of HA leads to a higher Young’s modulus for any percentage, reaching 22.0% at 15.0 wt% of HA. On the contrary, the tensile strength is higher with 5.0 wt% of HA (10.7%), while a noticeable drop is detected at 15.0 wt% of HA (26.7%). Strain at ultimate tensile strength (ε*) and toughness undergo a sharp downturn in all cases.

**Figure 5:**
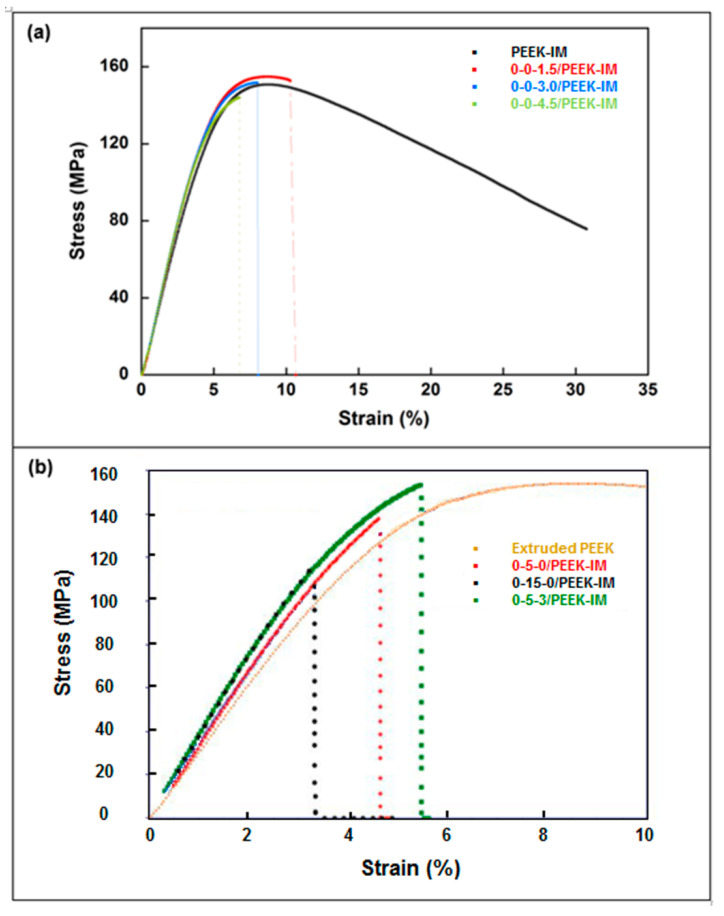
Bending test results for samples prepared by injection molding compared with virgin and extruded PEEK. (**a**) Influence of graphene nanoplatelet (GNP) wt% concentration: 0-0-xx/PEEK-IM, and (**b**) Influence of hydroxyapatite (HA) wt% concentration: 0-xx-0/PEEK and a composite manufactured with both kinds of filler (HA and GNP) at fixed concentrations: 0-5-3/PEEK. In all cases, the composites do not contain carbon fiber reinforcement.

Figure 5 and Table 5 show the results obtained from three-point bending tests. Results indicate that the incorporation of GNPs increases Young’s modulus with respect to extruded PEEK, reaching an increase of 11.5% at the highest concentration. Flexural strength drops by 5.3% only for the highest GNP concentration with respect to extruded PEEK. For an intermediate GPN concentration (3.0%), Young’s modulus increases by 10.2% and flexural strength is maintained. The addition of HA leads to an increase in Young’s modulus for any percentage, reaching 22.0% at 15.0 wt% of HA. In contrast, the flexural strength increases for 5.0 wt% of HA (4.6%), while a noticeable drop is detected at 15.0 wt% of HA (31.6%). Strain at ultimate tensile strength (ε*) undergoes a sharp downturn in all cases.

In view of the aforementioned results, the 0-5-3/PEEK formulation could be a priori a good composite respect to the tribological and osseointegration requirements for potential medical applications. However, although the Young’s modulus and strength are higher, both for tension and bending, than in extruded PEEK, they are not sufficient for medical devices in orthopedic devices. 

**Figure 6:**
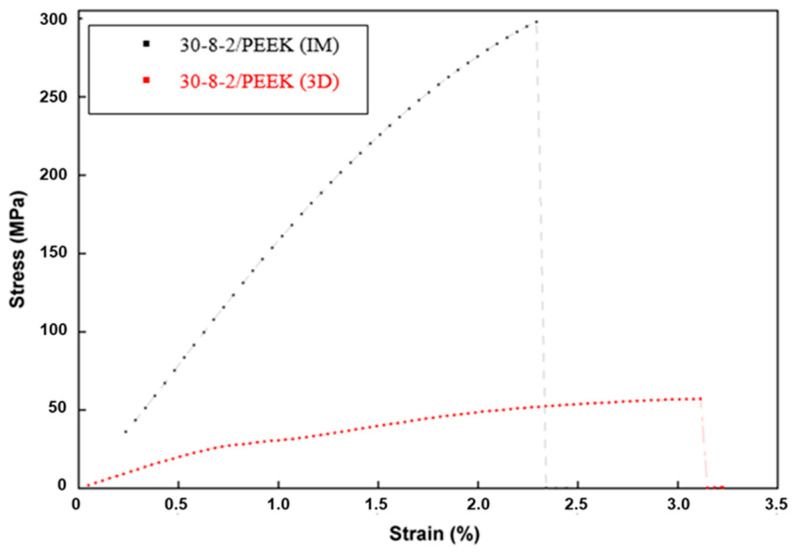
Bending test for 30CF-8HA-2GNP/PEEK composites prepared by injection molding and 3D printing.

On the contrary, the material reinforced using CFs increases the Young’s modulus and tensile stress in the case of the injected material (Table 6, Figure 6). Young’s modulus increased by 416.0% with respect to that of the extruded PEEK, while flexural strength increased by 94.1%. In the case of 3D-printed material, Young’s modulus was only 34.5% higher than that of the extruded PEEK, while flexural strength was 62.3% lower. Strain at ultimate tensile strength underwent a sharp downturn both for IM and 3D materials, reaching similar values.

According to the initial purposes of the article, i.e., to optimize the mechanical properties together with wear resistance and a better interaction with the bone, we incorporate also 8% of HA and 2% GNPs. The HA amount corresponds to an intermediate value between 5 and 15 wt% in order to take advantage of its osseointegration capability, and the 2 wt% GNP concentration is chosen because its presence as reinforcement in PEEK provided a 36% reduction in wear rate and 23% in coefficient of friction with respect to the neat PEEK, according to [22]. Both values are suitable for obtaining a low loss of mechanical properties. 

### 3.3. Osteoblast Viability Assays

**Figure 7:**
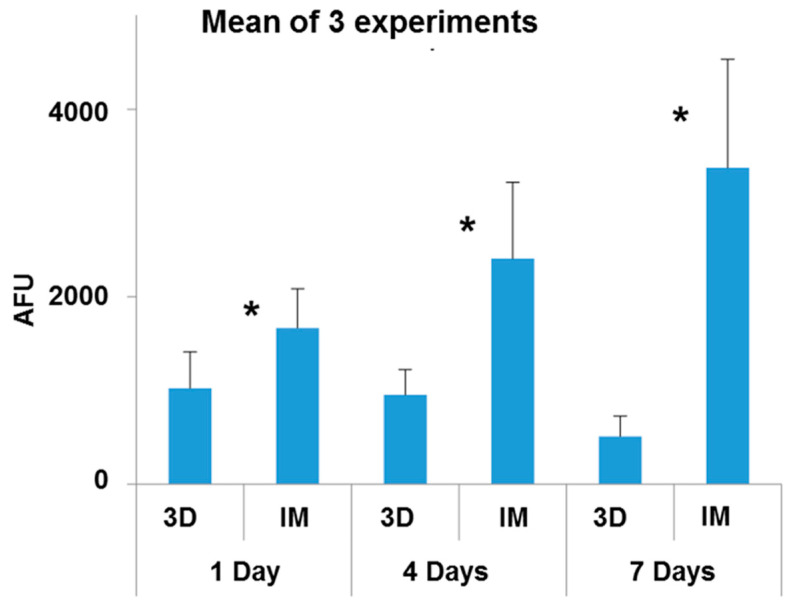
Viability of osteoblasts cultured for 1, 4 or 7 days on 30-8-2/PEEK-composites obtained by 3D printing (3D) and injection molding (IM). Results are expressed in arbitrary fluorescence units (AFUs). The mean ± SD is plotted. * Statistically significant (*p* < 0.05).

The viability of human osteoblasts cultured for 1, 4 or 7 days on 30-8-2/PEEK samples obtained by 3D printing or IM was evaluated. Three independent experiments were performed in duplicate. Figure 7 corresponds to the mean of the three experiments.

It was observed that the cells adhered to both surfaces tested after 1 day in culture, although the viability of osteoblasts was higher on samples obtained by IM than by 3D printing. The viability of osteoblasts cultured on IM samples increased with increasing culture time, while on samples obtained by 3D printing it decreased. At all the cultivation times tested, the cell viability was notably higher on samples obtained by IM than by 3D printing.

### 3.4. Cell Morphology and Actin Cytoskeleton Assays

**Figure 8:**
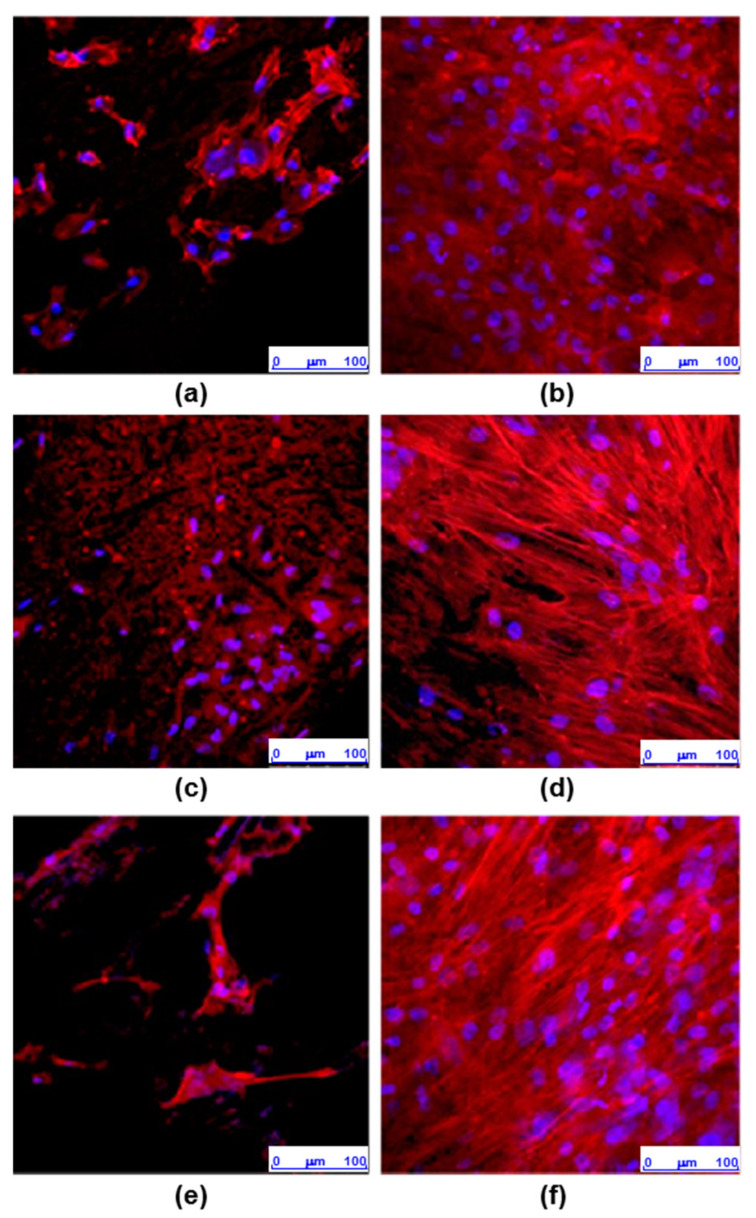
Images obtained by confocal microscopy of osteoblasts cultured for 7 days on 30-8-2/PEEK-composites samples obtained by 3D printing (3D) or injection molding (IM). Images corresponding to the maximum intensity projection of the double staining of actin (red) and nuclei (blue) are shown: (**a**) 3D printing, exp. 1; (**b**) injection molding, exp. 1; (**c**) 3D printing, exp. 2; (**d**) injection molding, exp. 2; (**e**) 3D printing, exp. 3; (**f**) injection molding, exp. 3.

The morphology of osteoblasts cultured for 7 days on 30-8-2/PEEK samples generated by 3D printing or IM were examined by staining the actin filaments and the cell nuclei. Three independent experiments were performed, with a single sample corresponding to each condition. Figure 8 shows a representative image of each experiment. In some cases, the materials stained nonspecifically with phalloidin-TRITC, interfering with the visualization of the cell cytoskeleton (Exp 1, sample IM, Exp 2, sample 3D). On both surfaces, nuclei were detected with normal morphology without signs of condensation. On the IM samples, the cells were arranged over the entire surface, showing an elongated morphology with the actin cytoskeleton organized into stress fibers distributed in parallel and mostly oriented along the longitudinal axis of the cell. In contrast, on the surfaces obtained by 3D printing, few adhered cells were observed, with poorly defined actin cytoskeletons and, for the most part, they formed small clusters.

## 4. Discussion

The contact angle in the 30-8-2/PEEK-IM composite, 79.7 ± 5.3°, was higher than the value of 75.6 ± 1.9° in pure PEEK [14], which is hydrophobic due to the presence of aromatic ring and polyester functional groups. The presence of carbon filler reinforcement increased the hydrophobic behavior value up to 91.3 ± 1.5° [32]. On the contrary, the incorporation of HA into the composite should help to increase the hydrophilic character due to its hydroxyl groups, as was observed by Ma et al. [14] compounding 30 wt% of HA into PEEK. A similar effect was caused by the graphene when this was incorporated into CFR/PEEK [32]. In our composite, the small amounts of HA and graphene seem to have been able to compensate for the increase in the contact angle due to the presence of CFs. On the other hand, the small increase found in the contact angle in the composite manufactured by 3D printing with respect to that formed by IM, correlates with the increase obtained in pure PEEK when this polymer is fabricated by the 3D additive manufacturing technique, the angle reaching θ = 84.6 ± 9.6° [5]. 

The influence of the manufacturing method is even more evident in the case of another surface property, namely, roughness. Notably, while the Ra of the IM composite is only several microns, since it reproduces the roughness of the internal cavity of the mold, the intrinsic characteristics of the 3D printing process generates a roughness of tens of microns. Another difference is the presence of great cracks in the 3D composite. The surface microscopical images also point out the presence of small holes in both cases. The first difference, i.e., the roughness, could influence the bioactivity of the composite, but the presence of the cracks could be another factor affecting the bioactivity, besides explaining the loss of toughness in the 3D specimen, which is more pronounced than in the composites obtained by injection molding. 

As regards mechanical properties, in the tensile tests, the addition of GNPs results in lower ultimate stress in the samples prepared by IM than in extruded PEEK; and the difference grows with the percentage of GNPs. On the other hand, the trend is not clear for Young’s modulus, which remains virtually constant for doses up to 3.0% GNP, but significantly increases for a dose of 4.5% GNP. The presence of HA leads to a slight increase in tensile stress for a dose of 5.0% HA, while there is a notable drop for a dose of 15% HA; by contrast, Young’s modulus notably increases with the presence of HA, independently of the dose. Ultimate strain and toughness undergo a sharp downturn in all cases.

In the case of bending tests, a continuous increase in Young’ modulus is observed with increasing GNP dose; in contrast, ultimate stress decreases as the dose of GNPs increases. The addition of HA again results in a slight increase in tensile stress for a dose of 5.0% HA, a notable drop being observed for a dose of 15% HA, while Young’s modulus increases for any dose. Ultimate strain undergoes a sharp downturn in all cases.

The sharp downturn observed in ultimate strain and toughness is related to the presence of inner pores and interfaces in the case of IM and the presence of large discontinuities in the case of 3D printing (Figure 6).

The aforementioned results are consistent with those reported in other studies [18,21] with respect to the impact of adding HA. The weak bonding between the HA particles and the PEEK matrix decreases the strength, but the greater stiffness of HA results in a composite material with a higher Young’s modulus. The effect of GNPs is less clear. In any case, the different composites tested provide mechanical properties (Young’s modulus and ultimate strength) that are insufficient for applications with heavy mechanical demands, such as femoral stems. 

The presence of 30% CFs improves the mechanical performance in IM samples, Young’s modulus increasing by as much as 416% and ultimate stress by as much as 109%. Nonetheless, the same concentration in 3D-printed samples led to an increase in Young’s modulus of only 34% and a 62% decrease in ultimate stress. Those results are consistent with those reported by other authors [33]. 

In general, the mechanical changes in composites are mainly influenced by the amount and distribution of the fillers, and the defects occur during the manufacturing process. The Young’s modulus, both tensile and flexural, is directly related to the intrinsic modulus of each constituent and, in the case of GNP and carbon fibers, also to their high aspect ratio, which provides large interfaces with the matrix. The reduction in yield stress is more related to a loss of ductility due to a reduction in the number of deformation modes than to changes in the crystalline phases and therefore in the lamellar thickness, which is a relevant factor in the yield stress of semicrystalline polymers.

In view of the previous results of the mechanical properties, the 30-8-2 chosen combination of fillers can be considered to be acceptable for the intended applications.

In any case, due to the limitations with the 3D-printed specimens concerning the presence of pronounced anisotropy affecting the union between the layers, further research would be required to optimize the printing process, i.e., conducting a detailed analysis of the different parameters affecting the process.

**Figure 9:**
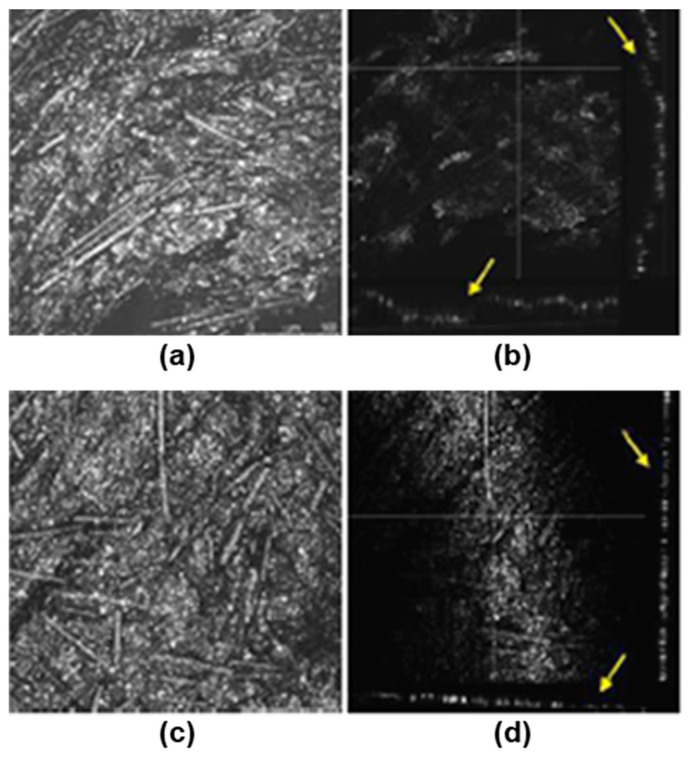
Images obtained by confocal microscopy of surfaces of PEEK-HA samples obtained by 3D printing or injection molding. Images corresponding to the maximum intensity projection and to orthogonal planes on the YZ and XZ axes (yellow arrows) are shown: (**a**) 3D printing maximum projection; (**b**) 3D printing orthogonal; (**c**) injection molding maximum projection; (**d**) injection molding orthogonal.

To investigate whether the differences in the cellular distribution on the composites obtained by 3D printing and IM could be related to changes in their topography, images of their surface were obtained by light reflection using a confocal microscope (Figure 2). In both types of samples, irregular surfaces with grooves were observed. The orthogonal plane images show the profile of the surfaces in the XZ and YZ axes and indicate that the surface roughness is much higher in samples processed by 3D printing than by IM (Figure 9).

The decrease in the viability of the osteoblast culture on the 3D-printed PEEK surface can be explained by the significant increase in surface roughness and the appearance of discontinuities. Both morphological surface factors may affect the regulation of cell growth in terms of extension, area, and volume, resulting in a less favorable surface for the cell. However, in spite of the roughness being the main surface property in both studied composites, it is difficult to distinguish the contribution of the crack to the cell interaction.

PEEK composites have been shown to promote cell attachment and proliferation, and osteogenic differentiation, and also to be associated with higher ALP activity and increased calcium nodule-formation, compared with pure PEEK using culture human osteoblast-like MG63 cells and murine fibroblast L929 cells obtained from ATCC [26]. Similarly, Wang et al. [27] reported that Sr/Si ion surface modification exhibits a better osteoinductive effect on the BMSCs derived from ovariectomized rats, improving PEEK osteointegration. Similar findings about PEEK osseointegration have been reported by other authors, with different cell lines or PEEK surface modifications [14,26,27]. 

## 5. Conclusions

- Two different PEEK composites were prepared using IM and a 3D printing technique based on the FFF process.
- The contact angle data indicated no significant differences in the surface wettability between the two types of surface, while the average roughness of the 3D printing sample was an order of magnitude higher than that of the sample obtained by IM. Confocal microscopy and SEM images showed the presence of small holes in both composites, with the appearance of strong cracks in the samples generated by FFF, which mostly affect the toughness behavior of the composite.
- Mechanical properties are strongly affected by the presence of any type of filler in the PEEK matrix. Ultimate strain and toughness undergo a sharp downturn, leading to brittle fracture behavior.
- The adhesion capacity of osteoblasts is higher on PEEK-HA surfaces obtained by IM than on those produced by 3D printing.
- The viability of osteoblasts grown on PEEK-HA surfaces obtained by IM improves by increasing the culture time up to 7 days, while it decreases on surfaces obtained by 3D printing.
- Osteoblasts cultured for 7 days on surfaces obtained by IM form a confluent monolayer, with an elongated morphology with a well-organized actin cytoskeleton, while on surfaces obtained by 3D printing the cells are arranged in isolated clusters showing less definite cytoskeleton.
- In summary, we can conclude that the CF-HA-GNP/PEEK surfaces obtained by IM allow the adhesion of human osteoblasts and their proliferation over time. In contrast, the surfaces generated by 3D printing, despite allowing the adhesion of osteoblasts, interfere with their growth, which could be explained by their topographic characteristics.

## Figures and Tables

**Table 1:** Composite material nomenclature. The three digits correspond to the weight percentages of the three potential reinforcement materials, CFs, HA and GNPs, followed by the term -IM for the samples manufactured by injection molding and -3D for the printed samples: xxCF-xxHA-xxGNP/PEEK-IM or xxCF-xxHA-xxGNP/PEEK-3D. (a) Composite materials without CF; (b) Composite materials with CF.

**(a)**						
	**Composite Material Nomenclature**					
**Component**	**0-0-1.5/PEEK-IM**	**0-0-3/PEEK-IM**	**0-0-4.5/PEEK-IM**	**0-5-0/PEEK-IM**	**0-15-0/PEEK-IM**	**0-5-3/PEEK-IM**
CF	0	0	0	0	0	0
HA	0	0	0	5	15	5
GNP	1.5	3	4.5	0	0	3
**(b)**						
	**Composite Material Nomenclature**					
**Component**	**30-0-0/PEEK-IM**		**30-8-2/PEEK-IM**		**30-8-2/PEEK-3D**	
CF	30		30		30	
HA	0		8		8	
GNP	0		2		2	

**Table 2:** Conditions tested in assays performed to assess osteoblast viability for the 30-8-2/PEEK composite, manufactured by injection molding (IM) and 3D printing (3D).

Sample	Incubation Period
3D samples + osteoblasts	1 day
IM samples + osteoblast	1 day
3D samples + osteoblasts	4 days
IM samples + osteoblasts	4 days
3D samples + osteoblasts	7 days
IM samples + osteoblasts	7 days

**Table 3:** Conditions tested in assays performed to assess cell morphology and organization of the actin cytoskeleton for the 30-8-2/PEEK composite, manufactured by injection molding (IM) and 3D printing (3D).

Sample	Incubation Period
3D samples + osteoblasts	7 days
IM samples + osteoblasts	7 days

**Table 4:** Tensile test results for samples prepared by injection molding compared with virgin and extruded PEEK. Samples correspond to the composites with different hydroxyapatite-graphene nanoplatelet (HA-GNP) wt% concentrations, but without carbon fiber reinforcement.

Material	E (GPa)	σ_ts_ (MPa)	ε* (%)	W (MJ/m^3^)
Virgin PEEK	3.9	68.8	41.9	41.9
Extruded PEEK	3.6 ± 0.2	89.5 ± 0.1	94.7 ± 4.3	65.0 ± 3.6
0-0-1.5/PEEK-IM	3.6 ± 0.2	85.8 ± 0.3	13.9 ± 0.9	9.15 ± 0.5
0-0-3.0/PEEK-IM	3.5 ± 0.1	82.5 ± 0.4	11.6 ± 0.4	7.33 ± 0.3
0-0-4.5/PEEK-IM	3.9 ± 0.3	79.6 ± 0.7	9.7 ± 0.2	5.76 ± 0.2
0-5-0/PEEK-IM	4.0 ± 0.1	99.1 ± 1.1	13.2 ± 1.9	10.2 ± 1.8
0-15-0/PEEK-IM	4.4 ± 0.5	65.6 ± 3.4	3.6 ± 0.2	1.3 ± 0.2
0-5-3/PEEK-IM	4.2 ± 0.2	90.8 ± 0.4	9.1 ± 0.1	6.0 ± 0.1

**Table 5:** Three-point bending test results for samples prepared by injection molding compared with virgin and extruded PEEK. Samples correspond to the composites with different hydroxyapatite-graphene nanoplatelet (HA and GNP) wt% concentrations, but without carbon fiber reinforcement.

Material	E_b_ (GPa)	σ_b_ (MPa)	ε* (%)
Virgin PEEK	3.1 ± 0.1	153 ± 1	31.5 ± 1.9
Extruded PEEK	3.1 ± 0.1	152 ± 2	30.3 ± 0.3
0-0-1.5/PEEK-IM	3.4 ± 0.1	155 ± 1	9.8 ± 0.8
0-0-3.0/PEEK-IM	3.4 ± 0.1	152 ± 1	7.9 ± 0.1
0-0-4.5/PEEK-IM	3.5 ± 0.1	144 ± 1	6.6 ± 0.2
0-5-0/PEEK-IM	3.5 ± 0.1	159 ± 10	7.0 ± 2.0
0-15-0/PEEK-IM	3.8 ± 0.1	104 ± 10	2.9 ± 0.3
0-5-3/PEEK-IM	3.8 ± 0.1	156 ± 2	5.8 ± 0.4

**Table 6:** Three-point bending test results for composites prepared by injection molding and 3D printing, compared to the virgin and extruded PEEK. Samples correspond to CF-0-0/PEEK and CF-HA-GNP/PEEK composites, with carbon fiber reinforcement.

Material	E_b_ (GPa)	σ_b_ (MPa)	ε* (%)
Extruded PEEK	3.1 ± 0.1	152 ± 2.0	30.3 ± 0.3
30-0-0/PEEK-IM	16.2 ± 0.3	319 ± 9.0	2.2 ± 0.1
30-8-2/PEEK-IM	16.1 ± 0.1	295 ± 8.0	2.3 ± 0.1
30-8-2/PEEK-3D	4.2	57.3	3.1

## Data Availability

Mechanical and surface characterization data is contained within the article. Bioactivity data available on request due to privacy and ethical reasons.
